# Differences in Applied Redox Potential on Cathodes Enrich for Diverse Electrochemically Active Microbial Isolates From a Marine Sediment

**DOI:** 10.3389/fmicb.2019.01979

**Published:** 2019-08-28

**Authors:** Bonita R. Lam, Casey R. Barr, Annette R. Rowe, Kenneth H. Nealson

**Affiliations:** ^1^Department of Biological Sciences, University of Southern California, Los Angeles, CA, United States; ^2^Department of Earth Sciences, University of Southern California, Los Angeles, CA, United States; ^3^Department of Biological Sciences, University of Cincinnati, Cincinnati, OH, United States

**Keywords:** extracellular electron transfer, electromicrobiology, cathode oxidation, iron oxidation, sulfur oxidation

## Abstract

The diversity of microbially mediated redox processes that occur in marine sediments is likely underestimated, especially with respect to the metabolisms that involve solid substrate electron donors or acceptors. Though electrochemical studies that utilize poised potential electrodes as a surrogate for solid substrate or mineral interactions have shed some much needed light on these areas, these studies have traditionally been limited to one redox potential or metabolic condition. This work seeks to uncover the diversity of microbes capable of accepting cathodic electrons from a marine sediment utilizing a range of redox potentials, by coupling electrochemical enrichment approaches to microbial cultivation and isolation techniques. Five lab-scale three-electrode electrochemical systems were constructed, using electrodes that were initially incubated in marine sediment at cathodic or electron-donating voltages (five redox potentials between −400 and −750 mV versus Ag/AgCl) as energy sources for enrichment. Electron uptake was monitored in the laboratory bioreactors and linked to the reduction of supplied terminal electron acceptors (nitrate or sulfate). Enriched communities exhibited differences in community structure dependent on poised redox potential and terminal electron acceptor used. Further cultivation of microbes was conducted using media with reduced iron (Fe^0^, FeCl_2_) and sulfur (S^0^) compounds as electron donors, resulting in the isolation of six electrochemically active strains. The isolates belong to the genera *Vallitalea* of the *Clostridia*, *Arcobacter* of the *Epsilonproteobacteria*, *Desulfovibrio* of the *Deltaproteobacteria*, and *Vibrio* and *Marinobacter* of the *Gammaproteobacteria*. Electrochemical characterization of the isolates with cyclic voltammetry yielded a wide range of midpoint potentials (99.20 to −389.1 mV versus Ag/AgCl), indicating diverse metabolic pathways likely support the observed electron uptake. Our work demonstrates culturing under various electrochemical and geochemical regimes allows for enhanced cultivation of diverse cathode-oxidizing microbes from one environmental system. Understanding the mechanisms of solid substrate oxidation from environmental microbes will further elucidation of the ecological relevance of these electron transfer interactions with implications for microbe-electrode technologies.

## Introduction

The discovery that microorganisms can uptake electrons supplied by a cathode (electrode poised at electron donating potentials) has significant ecological and industrial importance. Microorganisms with the ability to receive or donate electrons, extracellular electron transfer (EET), from insoluble substrates are integral players when it comes to the biogeochemical cycling of sulfur and iron (Emerson et al., 2010; Holmkvist et al., 2011; Shi et al., 2016). The capability of microorganisms to use insoluble substrates for energy could help explain why microbial life exists thousands of meters below the seafloor, and/or in some of the most oligotrophic regions of the world (Jørgensen and D’Hondt, 2006; D’Hondt et al., 2009; Hoehler and Jørgensen, 2013). Direct measurements of microbial metabolism in the deep dark ocean far exceed models based on the influx of organic carbon to the system (Burd et al., 2010) suggesting other sources of energy, such as reduced or conductive minerals, may be critical for microbial life in the deep subsurface. However, the biochemical basis of these metabolisms is not well understood, especially across diverse lineages or across diverse redox environments. Using electrochemical techniques, strides have been made to understand the potential for solid substate supported microbial metabolisms in diverse ecosystems (Strycharz-Glaven et al., 2013; Gregoire et al., 2014; Rowe et al., 2015; Dennis et al., 2016; Jangir et al., 2016; Lam et al., 2018; Yates et al., 2018). Few of these reports explored more than a single redox potential, which we found in our previous work has the capability to support more diverse microbial communities (Lam et al., 2018). How the specific cathode-oxidizing microorganisms and/or the mechanisms of electron uptake vary with redox potential in an environmental setting remain largely uncharacterized.

Though the use of poised potential electrodes for studying microorganisms is not new, the vast majority of studies to date have focused on using electrodes as a surrogate for a mineral reduction or anodic microbe-electrode interaction (Nealson and Rowe, 2016). The first report of microbial cathode oxidation was demonstrated with electrodes serving as the sole electron donor coupled to the reduction of nitrate to nitrite and to the reduction of fumarate to succinate by two *Geobacter* species (Gregory et al., 2004). Several taxa typically known to reduce minerals (e.g., *Geobacter* and *Shewanella*) have been shown to be capable of not only electrode reduction but electrode oxidation as well (Gregory et al., 2004; Rosenbaum et al., 2011; Rowe et al., 2018). Electron uptake has also been demonstrated in acetogenic bacteria (Nevin et al., 2011), iron-oxidizing bacteria (Summers et al., 2013; Bose et al., 2014), and methanogenic archaea (Cheng et al., 2009; Lohner et al., 2014; Deutzmann et al., 2015; Rowe et al., 2019), suggesting substantial phylogenetic diversity with regard to this metabolic capability. The diverse redox potentials used for these microbe-electrode interactions also suggests a great deal of mechanistic diversity. From an applied perspective, there has been an increased focus on utilizing microorganisms to catalyze the cathode reactions in microbial fuel cell (MFCs) systems (Clauwaert et al., 2007; Chen et al., 2008). Typically the most effective catalysts for cathodic reactions in MFCs have been materials (e.g., platinum) that are prohibitively expensive for large scale operation. Microbial mediated catalysis of cathodic reactions may provide an alternative to expensive abiotic catalysts with associated economic and environmental advantages in the generation of useful chemicals through cathodic biochemical synthesis (Rozendal et al., 2009; Huang et al., 2011). The field of microbial electrosynthesis, in which microorganisms utilize electrons from cathodes to produce organic carbon compounds is of particular interest due to the potential of sustainable product generation and synthesis of biofuels (Rabaey and Rozendal, 2010; Tremblay and Zhang, 2015). An increased understanding of what microorganisms or microbial communities are capable of cathode oxidation, how they are performing EET, and how activity relates to electrode redox potential will be necessary to optimize these potential industrial applications.

Most enrichments of cathode-oxidizing microorganisms have originated from applied systems such as wastewater or microbial fuel cells (Clauwaert et al., 2007; Rabaey et al., 2008; Wrighton et al., 2010). Natural environments with reduced, solid compounds (e.g., FeS, elemental sulfur) have been shown to be particularly good for enriching microorganisms capable of cathode oxidation (deCamposRodrigues and Rosenbaum, 2014). Our prior work was one of the few reports (Strycharz-Glaven et al., 2013) of an electrochemical enrichment of cathode-oxidizing microorganisms from an environmental system, specifically a marine sediment (Rowe et al., 2015). However, this work focused on a single applied potential. In order to investigate the ability for different redox potentials to support different microbial communities, in the same system we investigated how microbial community structure diversity and abundance relates to redox potential (Lam et al., 2018). As hypothesized, a more diverse microbial community was supported at more reducing electrode potentials, likely owing to the energetic favorability of coupling more reduced electrode potentials with a wider range of terminal electron acceptors (Lam et al., 2018). For example, *Desulfobacterales* and *Campylobacterales* which could be important players in electrode oxidation were enriched on lower potential electrodes. Here we further expand the repository of environmental isolates with cathode oxidation ability, and focus on conditions that provide more reducing electrode potentials. Our working hypothesis was that with a variety of redox potentials between −400 to −750 mV (vs. Ag/AgCl) and different terminal electron acceptors (nitrate and sulfate), we would be able to isolate a greater diversity of cathode-oxidizing organisms as has been suggested by our preliminary cathodic community investigation.

## Materials and Methods

### Three-Electrode Sediment Free Bioreactor Set-Up and Electrochemical Enrichment Operation

Working indium tin oxide (ITO) electrodes (1.5 cm × 7.5 cm; 11.25 cm^2^ surface area) from sediment microcosm incubation experiments of a previous study (Lam et al., 2018) along with electrodes from another microcosm enrichment conducted (June, 2015) were the source material for further electrochemical enrichment. Both sediment microcosm incubation experiments were conducted with sieved marine sediment collected from Catalina Harbor, CA (33.4285° N, 118.5090° W). Electrodes were stored and transferred in anaerobic stoppered bottles filled with artificial seawater (ASW) base after sediment microcosm incubation. ASW base was prepared as described (Rowe et al., 2015) and contained 342 mM NaCl, 14.8 mM MgCl_2_.6H_2_O, 0.1 mM CaCl_2_.2H_2_O, and 6.7 mM KCl. Nitrogen, phosphorous, and sulfur were added to the ASW base to yield concentrations of 10 mM NH_4_Cl, 1 mM KH_2_PO_4_ (pH 7.2), and 1 mM Na_2_SO_4_. NaHCO_3_ was added at a concentration of 5 mM to support autotrophic growth. The ASW media was utilized for all bioreactors and subsequent enrichments. The transferred electrodes served as the working electrodes in three-electrode sediment-free bioreactors used for enrichment of cathode-oxidizing microorganisms [illustrated in Supplementary Figure 1]. The bioreactors were autoclaved and all subsequent preparation was conducted in an anaerobic chamber maintained with a 10% H_2_, 5% CO_2_, 85% N_2_ gas mix and N_2_ purge gas (Coy Laboratory Products, Inc., Grass Lake, MI, United States) (Supplementary Figure 1B). The working electrodes were poised at the same redox potential they were set at during initial sediment microcosm incubation, ranging from −400, −500, −550, −650, and −750 mV vs. Ag/AgCl. Reference electrodes (Ag/AgCl in 3M NaCl, BASi, West Lafayette, IN, United States) and platinum counter electrodes were utilized in the three-electrode systems. Reference electrodes were sterilized with 70% ethanol and UV light prior to use. Each bioreactor contained 250 mL ASW media. Redox potential was controlled and applied using an eDAQ quad channel potentiostat (eDAQ Inc., Colorado Springs, CO, United States). Redox potential and current production were monitored and recorded with the eCorder eCHART software (eDAQ Inc., Colorado Springs, CO, United States) every 5 min.

The bioreactors were provided with either sulfate or nitrate to serve as the terminal electron acceptor (TEA). Geochemical analyses of the sediment microcosms and 16S rRNA community analysis of initial electrode biofilms from our previous work provided insight into sulfate and nitrate potentially being important TEAs coupled to electrode oxidation (Lam et al., 2018). With the addition of each TEA (final concentration of 200 μM Na_2_SO_4_ or NaNO_3_) electron uptake was monitored for changes in current, as negative current is indicative of cathode oxidation.

### Chemical Analyses of Bioreactors

During the course of electrochemical bioreactor enrichment, media from the bioreactors was removed and replaced with fresh sterile ASW media every 4 weeks. Removed media was analyzed for total proteins, anions, and pH. Media was filtered through 0.2 μm Acrodisc Supor^®^ membrane syringe filters (Pall Corporation, Port Washington, NY, United States) and filtrate was stored at −80°C until analysis for anions. Two mL aliquots of media were centrifuged at 13,000 rpm for 5 min to concentrate cells for total protein analysis. Pellets were stored at −20°C until analysis. To measure total proteins, pellets were hydrolyzed in 1 M NaOH at 50°C for 30 min with vigorous mixing by vortex at 10 min intervals. Soluble protein in the extracts was determined spectrophotometrically using Folin phenol reagent (Lowry et al., 1951) with bovine serum albumin (BSA) as a standard. Anion analysis was performed using a Metrohm Ion Chromatograph (Metrohm, Riverview, FL, United States) with an anions seawater separation column (Metrosep A SUPP 250, Metrohm). The ion chromatograph was run as per manufacturer protocol with 3.2 mM sodium bicarbonate, 1 mM sodium carbonate running buffer containing 2% acetonitrile at a flow rate of 0.7 mL/min.

### DNA Extraction and 16S rRNA Community Analysis of Bioreactor Communities

DNA extraction on electrode biofilms was conducted as previously described (Lam et al., 2018). Planktonic cells were collected by centrifuging 50 mL of media from the bioreactors at 10,000 rpm for 10 min at 4°C. Resultant cell pellets and electrode biomass were directly added to bead beating tubes from a MO BIO PowerSoil DNA Isolation Kit (Qiagen, Carlsbad, CA, United States). DNA extraction was performed using the MO BIO PowerSoil DNA Isolation Kit following manufacturer’s protocols. Extracted DNA quality and quantity was assessed using a Nanodrop (Thermo Fisher Scientific, Wilmington, DE, United States).

Illumina MiSeq paired-end (2 × 250 bp) sequencing was done on the V4 hypervariable region of the 16S rRNA gene of extracted DNA by MR-DNA (Molecular Research LP, Shallowater, TX, United States). In brief, ribosomal sequences were amplified with a 30-cycle PCR reaction with a 53°C annealing temp using barcoded 515F primer paired with 806R primer, and amplicons were pooled and purified with calibrated Agencourt Ampure XP beads (Agencourt Bioscience Corporation, Beverly, MA, United States). DNA libraries were prepared using the resultant PCR product with the Illumina TruSeq DNA library preparation protocol and sequenced on an Illumina MiSeq following manufacturer’s procedures (Illumina Inc., San Diego, CA, United States). Average sequence lengths of 250 bp and >70,000 sequences were obtained for all samples.

QIIME (Quantitative Insights Into Microbial Ecology) bioinformatics pipeline (Caporaso et al., 2010) was applied for barcode removal, quality filtering (Bokulich et al., 2013), and chimera checks (Edgar et al., 2011). *De novo* operational taxonomic unit (OTU) picking was utilized for taxonomy assignment based on 97% similarity and phylogenetic analysis was conducted using the Greengenes database (Werner et al., 2012). Downstream analyses of 16S rRNA sequencing data such as non-metric multidimensional scaling (NMDS) were run with the R package, Phyloseq (McMurdie and Holmes, 2013). All downstream analyses were based on normalization of reads (*n* = 74,182 sequences) of community samples to account for differences in sequencing depth. Evaluation of the contribution of different variables to bioreactor microbial community variation was done using permutational multivariate analysis of variance (PERMANOVA) with the Adonis function (Anderson, 2006).

### Enrichment Using Insoluble Electron Donor Media and Isolation

Various media containing insoluble electron donors were utilized to further enrich for cathode-oxidizing microorganisms. Iron was provided in the form of elemental granules (1–2 mm, 99.98% metals basis, Alfa Aesar, Haverhill, MA, United States). The iron granules served as the electron donor source in anaerobic seawater media supplemented with either nitrate or sulfate as the TEA. Media were prepared using standard anaerobic culturing techniques with N_2_ gas in the headspace of serum vials and tubes (Miller and Wolin, 1974). After several passages, dilution to extinction of Fe^0^/SO_4_ and Fe^0^/NO_3_ enrichments were conducted in FeCl_2_ agar shake tubes (1-3% agar). Elemental sulfur was prepared (Moser and Nealson, 1996) and added to ASW media to make agar plates with high (30 mM) and low sulfur (3 mM) concentrations (1.5% agar). The elemental sulfur agar plates were made with either nitrate or Fe(III)-NTA as TEAs. Difco marine broth (Difco, Lawrence, KS, United States) was also implemented as an enriched media to screen isolates for heterotrophic growth.

### Electrochemical Characterization of Isolates With Chronoamperometry and Cyclic Voltammetry

All electrochemical tests were conducted in three-electrode electrochemical cells (15 mL volume). ITO plated glass or carbon felt was used as the working electrode with counter and reference electrodes as described for sediment free bioreactors. Chronoamperometry was performed on isolates using the eDAQ quad channel potentiostat and eCORDER eCHART software as described above. Isolates were grown up overnight either in enriched marine broth (Difco, Lawrence, KS, United States) or ASW media supplemented with FeCl_2_ or S_0_ at 30°C. After 24 h, 20 mL of the overnight culture was pelleted at 13,000 rpm for 10 min and washed twice and resuspended in ASW base. Resuspended biomass was added directly to electrochemical cells sparged continuously with filtered air or nitrogen at a rate of 20 mL/min. If nitrate was used as the TEA, sodium nitrate was provided at a concentration of 200 μM nitrate in batch feeds. The electrochemical cells were either poised at + 100 mV (vs. Ag/AgCl) to initiate biofilm formation or directly exposed to cathode oxidizing conditions at −400 or −600 mV (vs. Ag/AgCl). Airflow was stopped and nitrogen was purged into the electrochemical cells at certain periods to see if electrode oxidation could be coupled to oxygen reduction. Furthermore, cyanide injections were done to look for inhibition in electrode oxidation. Cyclic voltammetry (CV) tests were conducted on the direct biofilms of isolates grown in the electrochemical cells or planktonic cells removed from the electrochemical cells to characterize the electrochemical activity of each fraction. CVs were performed with a Gamry Reference 600 potentiostat and Gamry Framework software (Gamry Instruments, Warminster, PA, United States). Each CV analysis was conducted at a scan rate of 5 mV/s over a range of −800 to +800 mV (vs. Ag/AgCl). Analysis on CV curves was performed with Gamry Echem Analysis (Gamry Instruments, Warminster, PA, United States) including calculation of mid-point potentials.

### Scanning Electron Microscopy (SEM) Analysis

Planktonic cells were filtered through sterile 0.2 μm Supor^®^ 200 filters (Pall Corporation, Port Washington, NY, United States). Filters and electrode samples were fixed in distillation purified electron microscopy grade 2.5% glutaraldehyde (Electron Microscopy Sciences, Hatfield, PA, United States). Samples were then processed through an ethanol dehydration series (30, 50, 70, 80, 90, 95, 100% v:v ethanol, three 10 min intervals for each concentration) and critical point drying (Autosamdri 815 critical point drier, Tousimis Inc., Rockville, MD, United States). Samples were placed on aluminum stubs and coated with Pd (Sputter Coater 108, Cressington Scientific, Watford, United Kingdom). Images were conducted at 5 keV using a JOEL JSM 7001F low vacuum field emission scanning electron microscope (JEOL USA, Inc., Pleasanton, CA, United States).

### Phylogenetic Analysis of Isolates

For taxonomic classification of isolates, stocks were streaked on enriched seawater agar plates (Difco, Lawrence, KS, United States) or agar plates made with FeCl_2_ or S_0_ as electron donor sources. PCR was performed directly on colonies or DNA extracted from pellets of overnight liquid cultures to amplify the 16S rRNA gene with the primers 27F and 1492R. Purified PCR product (DNA Clean & Concentrator^TM^-5, Zymo Research, Irvine, CA, United States) was sequenced (Genewiz, South Planfield, NJ, United States) with both primers. Sequences were quality checked using BioEdit (Carlsbad, CA, United States) and aligned against the SILVA database using the SINA aligner (v. 1.2.11) (Pruesse et al., 2012; Quast et al., 2013). Sequences from isolates from our prior work (Rowe et al., 2015) and nearest related organisms were also included in alignments. Maximum likelihood phylogenetic trees were built using the RAxML program (v. 8.1.11) (Stamatakis, 2014) with the ARB software package interface (Ludwig, 2004).

### Nucleotide Sequence Accession Numbers

16S rRNA sequences for bioreactor communities were deposited in the NCBI Sequence Read Archive (SRA) database (accession number: PRJNA503911). Isolate ribosomal 16S rRNA sequences have been deposited to GenBank (accession numbers: MK139821–MK139826).

## Results

### Bioreactor Electrochemical Enrichment and Current Production

**FIGURE 1:**
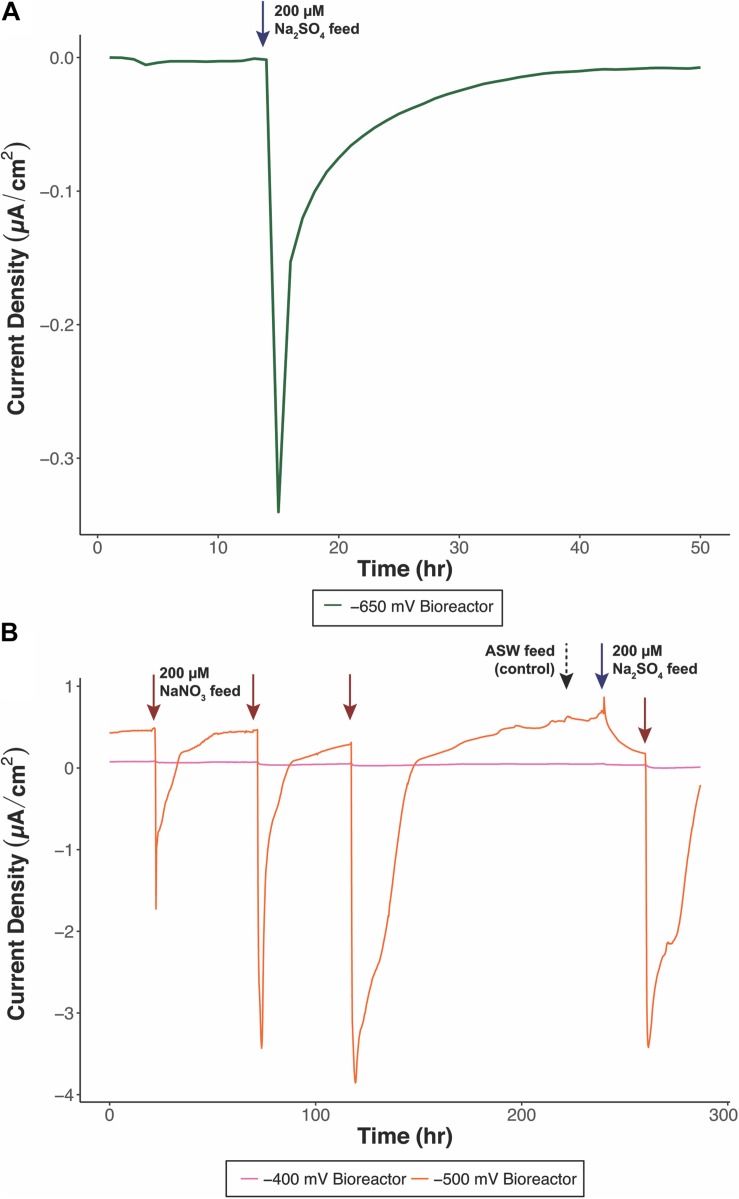
Representative chronoamperometry profiles of electrochemical bioreactors, **(A)** –650 mV (vs. Ag/AgCl) bioreactor fed with sulfate as TEA and **(B)** –400 mV (orange) and –500 mV (pink) (vs. Ag/AgCl) bioreactors fed with nitrate as the primary TEA. Blue arrows indicate sulfate feeds, red arrows indicate nitrate feeds, and the black dashed arrow indicates an artificial seawater base feed. Sulfate and nitrate additions correspond to a final concentration of 200 μM.

Five bioreactors were constructed and run with different redox potentials and electron acceptors (Table 1) for further enrichment of cathode-oxidizing microorganisms and determination of terminal electron accepting process. All bioreactors exhibited electron uptake by the cells (increases in negative current) when provided with a terminal electron acceptor (TEA). Typical responses in current in the bioreactors are demonstrated in Figure 1. There were no changes in current when bioreactors were not supplied with a TEA, suggesting that cathode oxidation only occurred when microorganisms had an available TEA to couple with electrode oxidation. In addition, electrode oxidation was highly dependent on the type of TEA provided to a given bioreactor, and minimal to no response was observed when alternative TEAs were provided (Figure 1B). Performance was evaluated as average Coulombic Efficiency (CE), which varied from 2.33% to 53.50% for the various reactors (Table 1).

**TABLE 1:** Summary of electrochemical bioreactor general run information.

**Cathodic Potential**	**Electron**	**Run Time**	**Average Coulombic**
**(mV vs. Ag/AgCl)**	**Acceptor**	**(days)**	**Efficiency*^a^***
−550	Sulfate	507	39.99%±3.56%
−650	Sulfate	293	4.09%±1.03%
−750	Sulfate	293	2.33%±1.94%
−400	Nitrate	573	4.99%±3.23%
−500	Nitrate	542	53.50%±5.88%

**^*a*^*Coulombic Efficiency values were calculated for the reduction of sulfate to sulfide for sulfate bioreactors (8 mol of electrons per mol of SO_4_) and for the reduction of nitrate to nitrite for nitrate bioreactors (2 mol of electrons per mol of NO_3_). Average Coloumbic Efficiency was calculated using all the electron acceptor feeds conducted during the course of bioreactor operation. Standard deviations of at least five Coloumbic Efficiency values shown from different electron acceptor addition time points.*

Media exchanges were performed consistently during operation. Measurements of pH, proteins, and anions were analyzed in media samples taken from bioreactors maintained for more than 300 days (−550, −400, and −500 mV vs. Ag/AgCl) (Table 2). A decrease in pH was observed for all bioreactors (pH of 7.43–7.91) compared to the ASW media’s initial pH of 8.2. Protein concentrations in the media had high variability during the run, ranging from 10.55 to 362.3 μg/ml. High protein concentrations generally occurred at points where negative current generation was the most robust (data not shown). Although each medium exchange resulted in the removal of the planktonic cells from the bioreactors, protein concentrations indicate that the planktonic communities were eventually re-established and therefore likely seeded from the electrode biofilm communities.

**TABLE 2:** Average pH, planktonic biomass proteins, nitrite and sulfate concentrations in media from electrochemical bioreactor media exchanges after 30 days of electrochemical incubation.

**Bioreactor**					
**Potential**					
**(mV vs.**	**Electron**		**Proteins**		
**Ag/AgCl)**	**Acceptor**	**pH**	**(μg/ml)**	**Anions**	
				**Nitrite**	**Sulfate**
				**(mM)**	**(mM)**
−550	Sulfate	7.91 ± 0.37	12.136*^a^*	ND*^b^*	0.04 ± 0.02
−400	Nitrate	7.49 ± 0.23	98.79 ± 118.51	0.49 ± 0.56	0.17 ± 0.06
−500	Nitrate	7.43 ± 0.23	88.28 ± 129.70	0.49 ± 0.67	0.19 ± 0.06

*Standard deviations of at least triplicate biological samples shown. During the course of 30 days approximately ∼1 mM of nitrate or sulfate was supplied to the bioreactors. *^*a*^*Denotes only one sample was obtained for measurement. *^*b*^*ND indicates not detected.*

Concentrations of nitrate, nitrite, and sulfate were measured as sulfate or nitrate served as the TEA for each bioreactor. Nitrate could not be detected in our samples and might have been present at levels below the limit of detection. The −550 mV (vs. Ag/AgCl) bioreactor was supplemented with 200 μM sulfate as the TEA, while the −400 and −500 mV (vs. Ag/AgCl) bioreactors were supplied with 200 μM nitrate as the TEA. During the course of a month between media exchanges, approximately 1 mmol of the corresponding TEA was added to each bioreactor. Sulfate was found in trace amounts in the −550 mV (vs. Ag/AgCl) bioreactor. Additionally, ASW media used for the bioreactors had a baseline concentration of 1 mM Na_2_SO_4_. The low levels of sulfate measured (0.04 mM) indicate the bioreactor communities were actively using sulfate as a TEA. The −400 and −500 mV (vs. Ag/AgCl) bioreactors also exhibited low levels of sulfate (0.17–0.19 mM) and nitrite in the 0.49 mM range. Nitrite measured in the bioreactor media could be a byproduct of nitrate reduction coupled to electrode oxidation. Other byproducts of denitrification (e.g., NO, N_2_O) were not measured, but could account for additional nitrate utilization. Although sulfate did not serve as the primary TEA for these bioreactors, there was a low level response in negative current when sulfate was added (Figure 1B). Other members of the bioreactor communities not actively engaging in cathode oxidation could also potentially use sulfate, though this was not explicitly investigated.

### Electrochemical Bioreactor Microbial Community Structure and Composition

**FIGURE 2:**
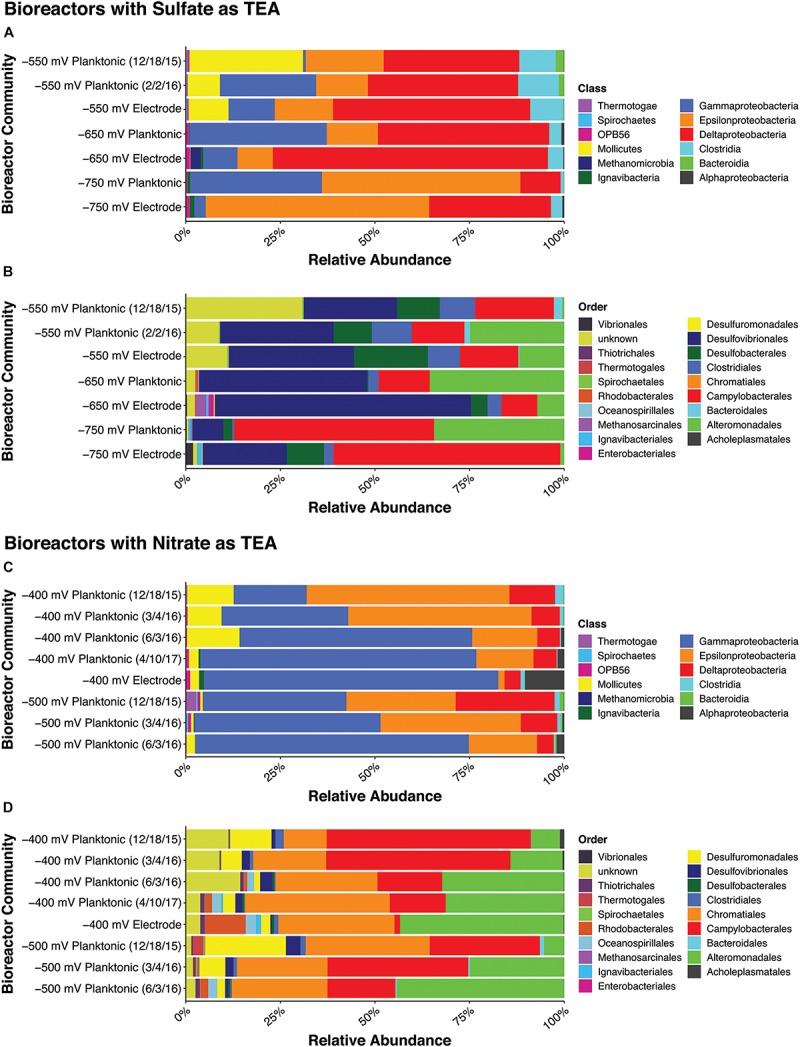
Relative abundances for each bioreactor community samples at the class (top 100 OTUs) and order (top 50 OTUs) level. The –550, –650, and –750 mV (vs. Ag/AgCl) sulfate electrochemical bioreactor communities are shown in **(A)** at the class level and in **(B)** at the order level. The –400 and –500 mV (vs. Ag/AgCl) nitrate bioreactor communities are shown in **(C)** at the class level and **(D)** at the order level.

Microbial community analyses were performed on final electrode biofilm communities and planktonic communities with amplicon next generation sequencing of the 16S rRNA gene. Shifts in community structure between redox potentials and mode of growth (planktonic vs. biofilm) were evaluated in terms of OTU abundance (Figure 2). For the bioreactors where sulfate served as the TEA (−550, −650, and −750 mV vs. Ag/AgCl), there was an overall increase in *Deltaproteobacteria* in the electrode biofilm communities and an increase in *Gammaproteobacteria* in the planktonic communities across all redox potentials. The most dominant members in the communities also changed with redox potential. At the class level, the −550 mV (vs. Ag/AgCl) bioreactor planktonic and biofilm communities had enrichments of *Mollicutes* (4.16%–15.29%) and *Clostridia* (4.34%–10.71%) that were not observed with the other redox potentials. The −750 mV (vs. Ag/AgCl) redox potential planktonic and biofilm communities had the highest overall enrichment of *Epsilonproteobacteria* (52.38% and 58.98% respectively). The −650 mV (vs. Ag/AgCl) redox potential bioreactor community had the greatest enrichment of *Deltaproteobacteria* (72.65%) along with a high abundance in the planktonic community as well (45.25%). The bioreactor communities where nitrate served as the TEA, −400 and −500 mV (vs. Ag/AgCl), were predominated by the *Gammaproteobacteria* compared to the sulfate bioreactors. The *Gammaproteobacteria* comprised 77.29–77.64% of the −400 mV (vs. Ag/AgCl) electrode biofilm communities. In comparison, the highest abundance of *Gammaproteobacteria* in the sulfate bioreactor communities was 36.29% in the −650 mV (vs. Ag/AgCl) planktonic community. We were unable to obtain an electrode biofilm sample for the −500 mV (vs. Ag/AgCl) bioreactor, because the electrode from that reactor experienced short-circuiting after 542 days of operation. The −400 mV (vs. Ag/AgCl) electrode communities exhibited a notable increase in the *Alphaproteobacteria* and to a lesser extent the *Gammaproteobacteria*. The −400 mV (vs. Ag/AgCl) planktonic communities had a greater abundance of the *Epsilonproteobacteria* and *Deltaproteobacteria* as compared to the electrode assemblages. Abundances at the order level for each set of bioreactors gave deeper insight into what specific microbial groups were enriched (Figures 2B,D). The *Campylobacterales* and *Alteromonadales* were consistently found across all redox potentials and communities. The sulfate bioreactor communities, which exhibited increases in the *Deltaproteobacteria*, had enrichments of the *Desulfovibrionales* (8.24–67.71%) and *Desulfobacterales* (2.32–19.50%). On the other hand, the communities in the nitrate bioreactors were highly enriched in the *Chromatiales* (11.36–38.92%).

**FIGURE 3:**
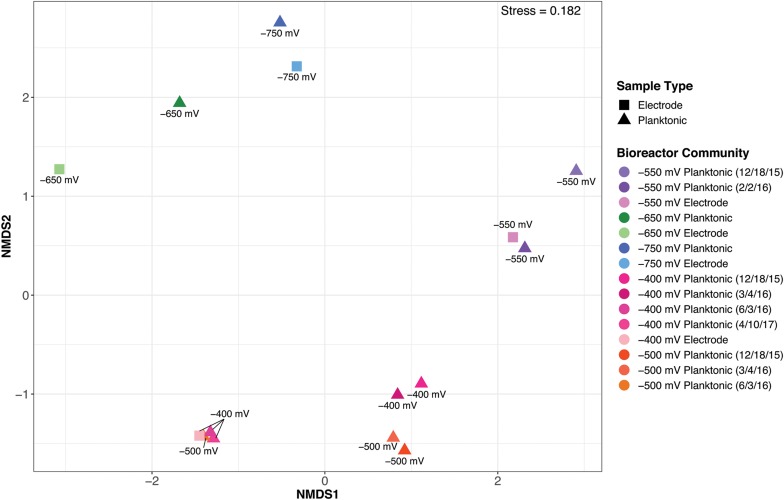
Bray-Curtis based non-metric multidimensional scaling (NMDS) plot of the electrochemical bioreactor community samples. Squares represent electrode biofilm samples and triangles signify planktonic samples.

Differences in overall community structure were quantified with hierarchical clustering using Bray-Curtis dissimilarity matrices (Figure 3). The NMDS plot demonstrates differences in microbial community structure with samples that are spatially closer together on the plot showing greater similarity. Overall, bioreactor communities from the same redox potential exhibited the closest similarity to each other. Although communities group together based on redox potential, the electrode biofilm and planktonic communities generally do not overlap. The exception to the aforementioned trend is with the −400 mV (vs. Ag/AgCl) redox potential communities. The −400 mV (vs. Ag/AgCl) electrode biofilm communities shared greater similarity to the later −400 and −500 mV (vs. Ag/AgCl) planktonic communities. A drastic shift in the planktonic communities of the −400 and −500 mV (vs. Ag/AgCl) bioreactors took place after the 3/4/16 sampling date. The more negative redox potentials, −650 and −750 mV (vs. Ag/AgCl) also spatially grouped together while the other redox potentials (−400, −500, and −550 mV vs. Ag/AgCl) were found to cluster closer together. Adonis tests were performed on weighted Unifrac values to determine the significance of variation observed in the microbial communities explained by growth mode (planktonic vs. biofilm), redox potential, and terminal electron acceptor. The mode of growth did not exhibit a significant impact on the variation between microbial communities. Redox potential contributed the most to variation (*R* = 0.71, *p* = 0.001), followed by the terminal electron acceptor used (sulfate or nitrate) (*R* = 0.44, *p* = 0.001).

### Isolation of Cathode-Oxidizing Strains and Confirmation and Characterization of Electrochemical Activity

**FIGURE 4:**
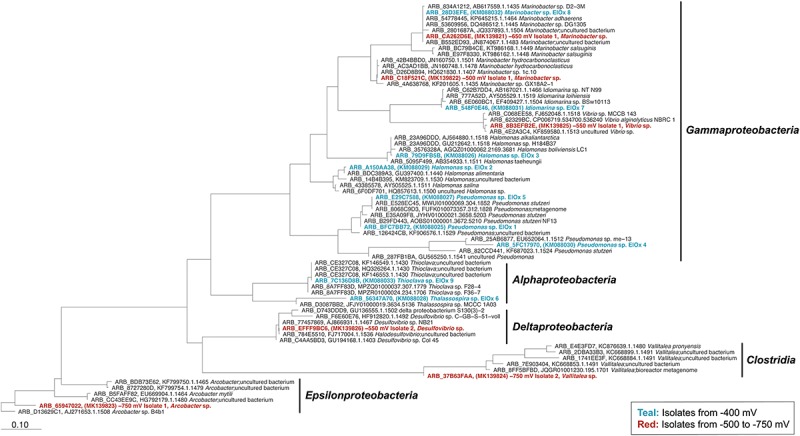
Phylogenetic tree constructed for all isolates based on full-length 16S rRNA gene sequences. The 16S rRNA sequences from isolates obtained from the pilot study are shown in teal (Rowe et al., 2015). The 16S rRNA sequences from strains isolated from this study are shown in red. The tree was constructed using the Randomized Axelerated Maximum Likelihood (RAxML) method. The scale bar indicates nucleotide substitutions computed using RAxML in ARB. GenBank accession numbers for isolates are indicated in parentheses. The *Arcobacter* sp. B4b1 (ARB_D13629C1) was selected as the tree root.

Isolation of pure cultures was approached by seeding secondary enrichment cultures with samples from the electrochemical bioreactors. Media were designed using iron (Fe^0^ and FeCl_2_) and sulfur (S^0^) as electron donor sources. Six isolates were obtained from subsequent culturing attempts using these media. The iron-oxidizing isolates obtained fell within the *Deltaproteobacteria* (*Desulfovibrio* genus), the *Clostridia* (*Vallitalea* genus), and the *Gammaproteobacteria* (*Marinobacter* genus) (Figure 4). The sulfur-oxidizing strains isolated are from the *Epsilonproteobacteria* (*Arcobacter* genus) and the *Gammaproteobacteria* (*Marinobacter* and *Vibrio* genera) (Figure 4). Isolates were also plated on enriched seawater agar plates to test for heterotrophic growth. All isolated *Gammaproteobacteria* strains (both *Marinobacter* spp. and the *Vibrio* sp.) were capable of growth on enriched marine media, and these strains were also capable of utilizing both nitrate and oxygen as electron acceptors. The *Deltaproteobacteria* strain (−550 mV Isolate 2) was capable of growth with both sulfate and nitrate, and the *Epsilonprotebacteria* (−750 mV Isolate 1) strain demonstrated growth with both nitrate and oxygen. The *Clostridia* strain (−750 mV Isolate 2) was only capable of growth with nitrate.

**FIGURE 5:**
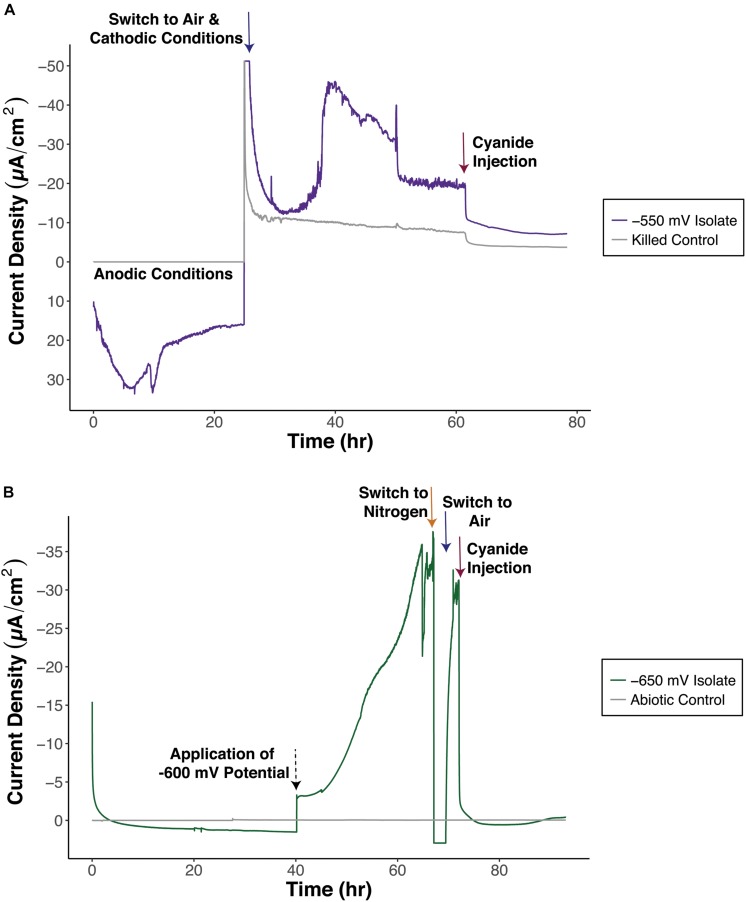
Representative chronoamperometry plots to test for electrochemical activity of isolates, **(A)** –550 mV Isolate 1, *Vibrio* sp. and **(B)** –650 mV Isolate 1, *Marinobacter* sp. –550 mV Isolate 1 was initially exposed to anodic conditions before switching to cathodic conditions. The applied potential for the –650 mV Isolate 1 was changed from –400 to –600 mV (vs. Ag/AgCl) at the 40 h time point. Arrows indicate switches in conditions. Blue arrows signify air and cathodic conditions, orange arrow indicates purging with nitrogen, red arrows indicate cyanide injection, and black dashed arrow indicates change in applied redox potential.

The electrochemical activity of isolates was confirmed with chronoamperometry. Isolates demonstrated cathode oxidation during chronoamperometry tests with oxygen or nitrate as a terminal electron acceptor (examples in Figure 5 and Supplementary Figure 2). In some cases, a slightly positive anodic potential (100 mV vs. Ag/AgCl) was initially applied to get strains to interact with the electrode before cathodic conditions were applied (Figure 5A). Strains were confirmed to perform electron uptake above the level of controls (sterile ASW media or autoclaved killed controls). In addition, for tests were oxygen was used as TEA, electrochemical cells were purged with nitrogen for periods of time to eliminate oxygen present. When no oxygen was provided, negative current ceased and dropped down to control levels (Figure 5B). Cyanide was also used to inhibit cytochrome *c* oxidase, preventing the transport of electrons from cytochrome *c* to oxygen (Figure 5). Negative current was no longer produced after cyanide was introduced to electrochemical cells. These results suggest a relationship between cathodic current and biological oxygen reduction. This adds to the collection of cathode-oxidizing organisms isolated from this site, that fell within the *Alphaproteobacteria* and *Gammaproteobacteria* from our prior work (Figure 4).

**FIGURE 6:**
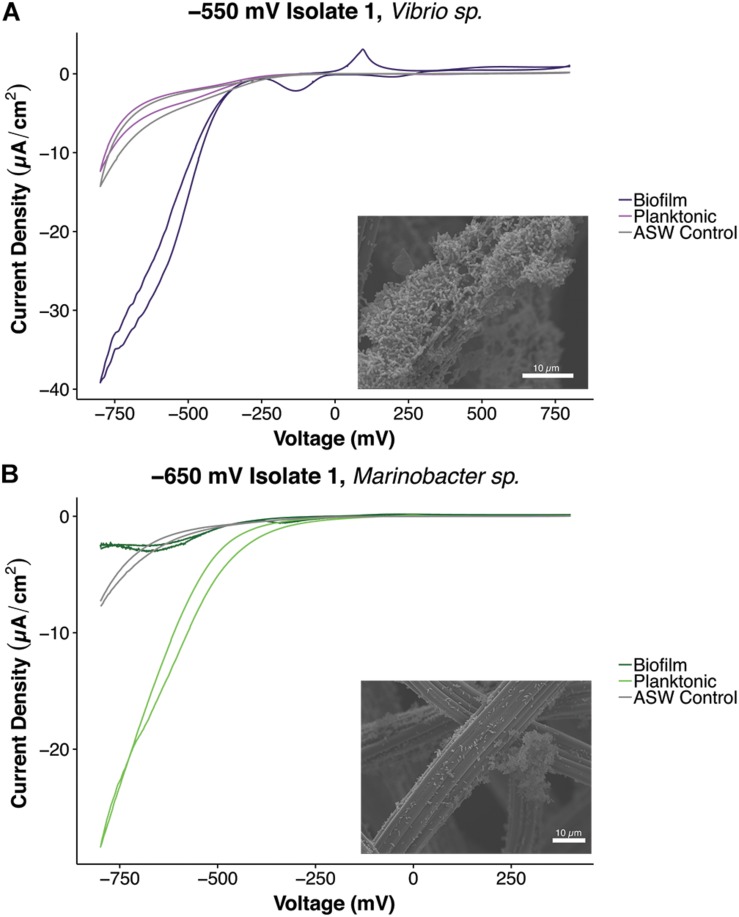
Plots of cyclic voltammetry tests conducted under non-turnover conditions of different cell fractions and scanning electron microscopic characterization of biofilms of isolates, **(A)** –550 mV Isolate 1, *Vibrio* sp. and **(B)** –650 mV Isolate 1, *Marinobacter* sp. Separate cyclic voltammetry curves were obtained for planktonic and biofilm fractions. The *Vibrio* strain exhibited electrochemical activity in the biofilm state, and the *Marinobacter* strain demonstrated electrochemical activity in both the planktonic and biofilm fractions. CV curves shown were the average curves obtained after running three cycles (curves) for all tests.

Investigation into the mode and potential mechanisms of EET conducted by the isolated strains was done with additional electrochemical tests (cyclic voltammetry) and imaging techniques. EET is currently known to occur through direct contact between the microbial cell and insoluble substrate (Myers and Myers, 1992; Reguera et al., 2005; Gorby et al., 2006; Kim B. et al., 2006; Clarke et al., 2011; Pirbadian et al., 2014) or through mediated processes involving shuttles that move electrons to and from the microbial cell to an insoluble substrate (Newman and Kolter, 2000; Marsili et al., 2008; von Canstein et al., 2008; Okamoto et al., 2013). Cyclic voltammetry tests were conducted on biofilms and planktonic cells to assay for electrochemical activity in each fraction (Figure 6 and Supplementary Figure 3). Analysis of CV plots revealed what fractions contributed to electrochemical activity by identifying peaks suggestive of oxidation or reduction (Figure 6). Midpoint potentials of the redox activity peaks were calculated (Table 3). For all strains, redox peaks indicative of flavins and other characterized electron shuttles were not detected. A wide range of midpoint potentials was obtained, with the iron-oxidizing isolates possessing more reducing midpoint potentials (−66.86 to −389.1 mV vs. Ag/AgCl) compared to sulfur-oxidizing isolates (99.20 to −59.4 mV vs. Ag/AgCl) (Table 3 and Supplementary Figure 3). Electrochemical activity was determined to come from biofilm/attached cells for all isolates. Additionally, both *Marinobacter* isolates also demonstrated electrochemical activity in the planktonic state. SEM imaging of electrodes revealed attached cells and biofilms on the electrode materials (Figure 6).

**TABLE 3:** Calculated midpoint potentials of isolates determined by cyclic voltammetry tests in planktonic and biofilm associated biomass.

	**Enrichment**	**Form of**	
	**Electron**	**Electrochemical**	**Midpoint Potential**
**Isolate**	**Donor**	**Activity**	**(mV vs. Ag/AgCl)**
−500 mV Isolate 1, *Marinobacter* sp.	Elemental Sulfur	Planktonic, Biofilm	35.09 ± 7.07
−650 mV Isolate 1, *Marinobacter* sp.	Elemental Iron	Planktonic, Biofilm*^a^*	−389.1 ± 34.65
−550 mV Isolate 1, *Vibrio* sp.	Elemental Sulfur	Biofilm	−59.40 ± 19.76
−550 mV Isolate 2, *Desulfovibrio* sp.	Elemental Iron	Biofilm	−202.65 ± 36.84
−750 mV Isolate 1, *Arcobacter* sp.	Elemental Sulfur	Biofilm	99.20 ± 8.49
−750 mV Isolate 2, *Vallitalea* sp.	Elemental Iron	Biofilm	−66.86 ± 36.69

*Standard deviations of technical triplicate tests shown from the same electrochemical cell. *^*a*^*Denotes a weak but quantifiable signal.*

## Discussion

Our bioreactor electrochemical enrichments allowed simultaneous simplification of the system to enrich for cathode-oxidizing microorganisms and the ability to continuously monitor electron uptake activity. This also allowed us to enrich for a specific TEA to be utilized by the bioreactor communities and screen for specific cathode oxidizing activity. Average Coulombic Efficiency values were calculated as way to evaluate electron capture from the electrode to the supplied TEAs. Consequently, the bioreactors operated for over 500 days had the highest CE values (up to 53.5%). Lower CE values (2.33–4.09%) were observed in the bioreactors where negative current diminished with time and thus operation of these bioreactors did not go for as long. The bioreactors with lower CE were run at the most negative redox potentials (−650 and −750 mV vs. Ag/AgCl) where hydrogen sulfide production could have impacted and inhibited members of the cathode assemblages (Reis et al., 1992; Caffrey and Voordouw, 2010; Delgado Vela et al., 2018; Kushkevych et al., 2019). After electrochemical enrichment, certain groups of organisms such as the *Alteromonadales* and *Campylobacterales* were found across all bioreactor samples despite differences in redox potential and TEA. In our prior investigation, the *Alteromonadales* were present in all enriched cathode-oxidizing microbial communities although their abundance varied with applied redox potential (Lam et al., 2018). Within the *Alteromonadales*, many strains have been characterized to be capable of EET including several *Shewanella* spp. (Fredrickson et al., 2008) along with *Idiomarina* sp. and *Marinobacter* spp. previously isolated by our group from the same site (Catalina Harbor sediments) (Rowe et al., 2015). With our prior work, the *Campylobacterales* were only observed when specifically analyzing OTU contributions to the presence of nitrate reductase in cathode-oxidizing communities (Lam et al., 2018). Further enrichment and selection of nitrate as a TEA, could explain the increased abundances of *Campylobacterales* in the electrochemical bioreactors. In addition, members of the *Campylobacterales* are known to utilize a diversity of electron acceptors including several sulfur species, arsenate, selenate, and Fe(III) (Finster et al., 1997; Stolz et al., 1999; Campbell et al., 2001). The *Campylobacterales* are an order within the *Epsilonproteobacteria*. *Epsilonproteobacteria* are commonly found in deep-sea hydrothermal vent and subsurface systems and are known for their chemolithotrophic metabolisms (López-García et al., 2003; Takai et al., 2005; Campbell et al., 2006; Yamamoto and Takai, 2011). *Epsilonproteobacteria* have been found to colonize the surfaces of carbon steel and stainless steel substrates submerged in coastal seawaters (Bermont-Bouis et al., 2007; Jones et al., 2007). Specifically OTUs within the *Campylobacterales* were observed in clone libraries of biocorroding microbial communities found on carbon steel slides after laboratory incubation in Pacific Ocean coastal seawaters (Dang et al., 2011). Additionally, members of the *Campylobacterales* were highly abundant in microbial communities found on working electrodes poised at 218 mV (vs. SHE) of bioelectrochemical systems inoculated with hydrothermal chimney samples (Gartman et al., 2017).

The effect of supplied TEA resulted in differences in the predominant groups enriched in sulfate and nitrate bioreactors. For the sulfate fed bioreactors (−550, −650, and −750 mV vs. Ag/AgCl), the enriched electrode biofilm communities showed a greater abundance of *Deltaproteobacteria* compared to planktonic communities. *Deltaproteobacteria* have been shown to be strongly associated with the anode electrodes compared to solutions in MFC systems (Ishii et al., 2014). The predominant mechanisms of EET characterized in *Deltaproteobacteria* involve direct contact between the microbial cell and insoluble substrate (Lovley et al., 2004; Logan, 2009). The predominance of *Deltaproteobacteria* in electrode communities was not observed for the −400 mV vs. Ag/AgCl bioreactor, which utilized nitrate as the primary TEA. Electrode biofilm communities from the nitrate fed bioreactor (−400 mV vs. Ag/AgCl) exhibited significant enrichments of the *Rhodobacterales* and *Chromatiales* not seen in the sulfate fed bioreactors. *Chromatiales* OTUs have been identified to be dominant members of denitrifying cathodes from fuel cell systems (Wrighton et al., 2010). Recently, a member of the *Chromatiales* was shown to be an important constituent of a biocathode enriched from seawater with studies suggesting it performs CO_2_ fixation driven by cathode oxidation (Leary et al., 2015; Wang et al., 2015; Yates et al., 2016). The applied redox potential (electron donor source) was the other primary driving factor of enriched bioreactor community structure. The −550 mV vs. Ag/AgCl bioreactor communities showed greater similarity to the −400 and −500 mV vs. Ag/AgCl bioreactor communities although they differed in TEA. This could be explained by the fact that the −550 mV vs. Ag/AgCl redox potential was not as energetically favorable to be coupled to the chosen TEA process (sulfate reduction) compared to the other sulfate bioreactor redox potentials (−650 and −750 mV vs. Ag/AgCl). The −550 mV vs. Ag/AgCl bioreactor communities thus more closely resembled the −400 and −500 mV vs. Ag/AgCl microbial communities with a decrease in abundance of strict sulfate reducers and an increase in taxa with greater metabolic flexibility (e.g., ability to use nitrate and sulfate as TEA). The mode of growth (planktonic vs. biofilm) did not significantly impact microbial community structure. Most studies of microbial communities from bioelectrochemical systems only characterize electrode attached communities, and investigations that look at both attached and planktonic communities primarily utilize microbial fuel cell systems. Differences in anode electrode biofilm communities and planktonic communities were reported in MFCs inoculated with activated sludge (Kim G.T. et al., 2006) and lagoon sediments (Ishii et al., 2014). Other studies report similarities in overall composition between planktonic and attached electrode communities (Xing et al., 2009), although one investigation also noted higher protein concentrations and rRNA gene copy number in planktonic communities (Shimoyama et al., 2009). Electrochemical enrichments using subsurface well waters as the initial source inoculum observed close similarities between planktonic and attached working electrode communities at the family level (Jangir et al., 2016). Similarities in composition between attached and planktonic communities suggest that the planktonic communities may have also been able to utilize the electrode as an energy source, perhaps though electron shuttling mechanisms of EET. An electron shuttle, flavin mononucleotide was detected in nM amounts in media removed from the bioreactors during operation (data not shown). Additionally, both of the *Marinobacter* strains isolated in our study demonstrated electrochemical activity in the planktonic state. Enhanced EET in microbial communities has been demonstrated with the addition of exogenous electron shuttles (Aulenta et al., 2011; Yamamura et al., 2018) or strains that generate endogenous shuttles (Pham et al., 2008). Endogenously produced electron shuttles have been measured from communities performing metal reduction (Fuller et al., 2014) and in bioelectrochemical systems (Uria et al., 2017). Future studies looking at microbial communities in bioelectrochemical systems should consider evaluating planktonic communities as these communities may be essential in supporting and contributing to electrochemical activity.

The use of diverse starting redox potentials led to the subsequent isolation of six new electrochemically active strains. All of the isolates obtained fall within the major groups that were enriched during electrochemical bioreactor operation. Our initial study resulted in the successful isolation of several electrochemically active strains in the *Alphaproteobacteria* and *Gammaproteobacteria* (Rowe et al., 2015). With this work, we have expanded the diversity of isolates into the *Epsilonproteobacteria*, *Clostridia*, and *Deltaproteobacteria*. We note that each of these lineages was distinctly enriched on the −550, −650, and −750 mV vs. Ag/AgCl incubations. An *Arcobacter* strain, −750 mV Isolate 1 was obtained through the sulfur-oxidizing enrichments. Several *Arcobacter* OTUs were found to colonize iron sulfide minerals incubated around Catalina Island (Ramírez et al., 2016) which was the source of the marine sediments utilized in our initial electrode enrichments. Electrochemical activity has only been described in one *Arcobacter* strain, *Arcobacter butzleri* ED-1, which was shown to donate electrons to an electrode (Fedorovich et al., 2009). This strain was isolated from a microbial fuel cell inoculated with marine sediment, and a proteomic investigation revealed differential expression in FlaA, a flagellin and two novel putative *c*-type cytochromes in anode associated cells compared to cells grown in normal batch culture (Pereira-Medrano et al., 2013). Genomic sequencing and investigation of our isolate would be necessary to evaluate the presence of these novel putative c-type cytochromes. There is much to be learned about how members of the *Arcobacter* genus perform EET as we are just beginning to obtain electrochemically active isolates within the *Epsilonproteobacteria*.

In this study, we report the first instance of an electrochemically active isolate from the genus *Vallitalea* (−750 mV Isolate 2, *Vallitalea* sp.) within the order *Clostridiales* that was isolated on reduced iron and exhibited electrochemical activity with nitrate as a TEA. There have been several reports of other members of the *Clostridia* exhibiting EET activity (Köpke et al., 2010; Wrighton et al., 2011; Choi et al., 2012, 2014), but the *Vallitalea* is a poorly described genus with no reports of electrochemical activity. Two recognized species of the genus were both isolated from hydrothermal systems, marine sediments from the Guaymas basin (Lakhal et al., 2013) and a chimney from Prony Bay in New Caledonia (Aissa et al., 2014). Recently, another *Vallitalea* strain was isolated from sediments collected from the Okinawa Trough (Sun et al., 2019). Further characterization of the *Vallitalea* isolate in this study is essential to expand our understanding of this genus and its potential for EET as it is commonly found in various distinct environments including in association with corals (Pootakham et al., 2017) and insect guts (Borrelli et al., 2017). Recently, a flavin-based mechanism of EET in *Listeria monocytogenes* was characterized, and orthologs of the genes responsible for EET in *L. monocytogenes* were identified in other species within the *Firmicutes* phylum (Light et al., 2018). Although this flavin-based mechanism was characterized under anode reduction conditions, the potential role of flavins to facilitate EET should be evaluated in our *Vallitalea* isolate.

One isolate from the *Deltaproteobacteria* was obtained, −550 mV Isolate 2 (*Desulfovibrio* sp.). Members of the *Desulfovibrio* genus are found in a wide variety of environments and are generally characterized as sulfate reducers (Brenner et al., 2001). Although the direct role of sulfate reducing bacteria in metal corrosion is a controversial topic, there have been studies that suggest sulfate reducing bacteria are capable of directly taking up electrons from iron (Dinh et al., 2004; Enning et al., 2012; Venzlaff et al., 2013). Some of the precipitates formed on the elemental iron used in Fe^0^/SO_4_ enrichment cultures (Supplementary Figure 4) resembled the mineral crust (FeS, FeCO_3_, Mg/CaCO_3_) described as being formed as a result of electron uptake from iron by *Desulfopila corrodens* strain IS4 and *Desulfovibrio ferrophilus* strain IS5 (Enning et al., 2012; Venzlaff et al., 2013). Transcriptomic analyses of *D. ferrophilus* IS5 identified outermembrane cytochromes that might be involved in electron uptake (Deng et al., 2018). Most recently, a study reported nitrogen assimilation by *D. ferrophilus* IS5 during electron uptake, indicating energy acquisition is coupled to electrode oxidation for this strain (Deng and Okamoto, 2018). In addition, in an investigation screening for cathode oxidation ability in several environmental isolates, production of cathodic current by *Desulfovibrio piger* was demonstrated (deCamposRodrigues and Rosenbaum, 2014). Other potential proteins involved in electron uptake for the *Desulfovibrio* genus have been suggested through metagenomic studies of syntrophic consortia of anaerobic methanotrophic archaea and sulfate reducing bacteria (Skennerton et al., 2017). The process of electron uptake from insoluble substrates by the *Desulfovibrio* genus could be of critical importance in explaining metal biocorrosion and microbial metabolism in various environments like the deep subsurface.

The two *Marinobacter* strains isolated came from different sources (−500 and −650 mV vs. Ag/AgCl bioreactors) and different subsequent enrichment culture electron donors (sulfur and iron, respectively). They also exhibited very different midpoint potentials, with the iron-oxidizing isolate possessing a significantly more reducing midpoint potential (−389.1 mV) compared to the sulfur-oxidizing isolate (35.09 mV). The two *Marinobacter* strains from our initial study were both isolated from FeS and had midpoint potentials between −176.2 and −184 mV (Rowe et al., 2015). These results suggest great metabolic flexibility and potentially diverse mechanisms of EET in the *Marinobacter* genus. The *Marinobacter* genus is ubiquitous in marine environments, and some *Marinobacter* spp. are known neutrophilic Fe(II) oxidizers (Edwards et al., 2003; Bonis and Gralnick, 2015). Genomic analysis of *Marinobacter aquaeolei* VT8 revealed the presence of 47 genes encoding cytochrome proteins (Singer et al., 2011). Outermembrane multiheme *c*-type cytochromes are known to be involved in EET for some of the most well studied and characterized electrogenic bacteria (Myers and Myers, 1992; Kim B. et al., 2006; Paquete and Louro, 2010). Additionally, a member of the *Marinobacter* was shown to be a predominant constituent of a biocathode derived from marine sediment (Strycharz-Glaven et al., 2013). Our *Marinobacter* strains are prime candidates for further investigation of the mechanisms of cathode oxidation in this metabolically versatile genus. A *Vibrio* strain, −550 mV Isolate 1, was another *Gammaproteobacteria* sulfur-oxidizing isolate obtained. *Vibrio* strains isolated from lake sediments have been shown to reduce Fe(III) and Mn(IV) (Jones et al., 1983, 1984a, b). Additionally, strains of the marine bacterium, *Vibrio natriegens*exhibited enhanced corrosion of stainless steel (Nivens et al., 1986; Cheng et al., 2010). Mechanisms of EET in the *Vibrio* genus have not been characterized, although there was a study that demonstrated *Vibrio parahaemolyticus*, a pathogen with an MtrB (outermembrane cytochrome protein identified in *Shewanella oneidensis* MR-1) homolog was capable of reducing insoluble iron and manganese (Wee et al., 2014). In addition, the *mtrCAB* gene cluster involved in the metal reduction pathway of *Shewanella* spp. was identified in the genomes of several *Vibrio* strains (Shi et al., 2012). With our *Vibrio* isolate, anodic current was produced before switching over to cathodic conditions suggesting the strain is also capable of electrode reduction. The observed homology in genes involved in metal reduction between *V. parahaemolyticus* and *S. oneidensis* MR-1, provides a starting point to investigate potential mechanisms of EET in our *Gammaproteobacteria* isolates.

Electrochemical characterization of the isolates highlighted the diversity in the dominant redox potential of the proteins that interact with the electrode (demonstrated by calculated midpoint potentials). Generally, a trend toward more reducing midpoint potentials for iron-oxidizing isolates (−66.86 to −389.1 mV) compared to sulfur-oxidizing isolates (−59.40 to 99.20 mV) was observed. In addition, isolates demonstrated electrochemical activity close to the redox potentials at which they were initially enriched (e.g., −650 mV Isolate 1 exhibiting electroactivity at an applied potential of −600 mV and not −400 mV). Although our isolated strains captured more diversity in the type of genera obtained, we still did not attain isolates from groups that might have contributed to cathode oxidation in the electrochemical bioreactors. For example, the *Chromatiales* were highly enriched in the −400 and −500 mV vs. Ag/AgCl electrochemical bioreactor communities, but were not captured when culturing with our designed enrichment media. This is consistent with other groups that have attempted isolation from a cathodic organism in this group (Eddie et al., 2016). New and innovative electrochemical isolation techniques must be explored to target groups of interest rather than traditional isolation techniques.

Using five different redox potentials, this work was able to enrich for six novel electrochemically active isolates from a single stratified marine sedimentary system. These isolates have further expanded the diversity of microorganisms capable of cathode oxidation. It is becoming more evident that microorganisms that are able to perform anode reduction are also capable of cathode oxidation (Gregory et al., 2004; Rosenbaum et al., 2011; Rowe et al., 2018). Two of the isolates obtained in this study come from genera (*Arcobacter* and *Vibrio*) where insoluble substrate reduction has been shown with no record of cathode oxidation. Investigating bacteria that are traditionally known as metal reducers for cathode oxidation might reveal the flexibility to also oxidize insoluble substrates is relatively common and explain the prevalence of some of these organisms in oligotrophic environments (Walsh et al., 2016) and environments with fluctuating redox gradients. Expanding electrochemical techniques to probe environments where reduced minerals predominate will help further the understanding of these metabolisms. It will be important to include the effects of electrode redox potential for capturing the scope of the electro-active community in future work. Sequencing and analysis of the genomes of these isolates will also provide valuable insights into the potential mechanisms being utilized to perform electron uptake. Insoluble substrate oxidation is an ability that seems to be widespread, though we are only beginning to understand the extent of this process in nature.

## Data Availability

The datasets generated for this study can be found in the NCBI Sequence Read Archive (Accession Number: PRJNA503911), GenBank (Accession Numbers: MK139821–MK139826).

## Author Contributions

KN, AR, and BL conceived and designed the work. BL and CB executed the experiments. BL, AR, and KN analyzed the data. BL drafted the manuscript. All authors wrote and revised the manuscript.

## Conflict of Interest Statement

The authors declare that the research was conducted in the absence of any commercial or financial relationships that could be construed as a potential conflict of interest.
