# Supplementary material for: Differences in Applied Redox Potential on Cathodes Enrich for Diverse Electrochemically Active Microbial Isolates From a Marine Sediment
Source: Front Microbiol. 2019 Aug 28;10:1979. doi: 10.3389/fmicb.2019.01979 (PMC6724507; doi:10.3389/fmicb.2019.01979)
Supplement: Supplementary file 1 [file Data_Sheet_1.pdf]

Supplementary Material

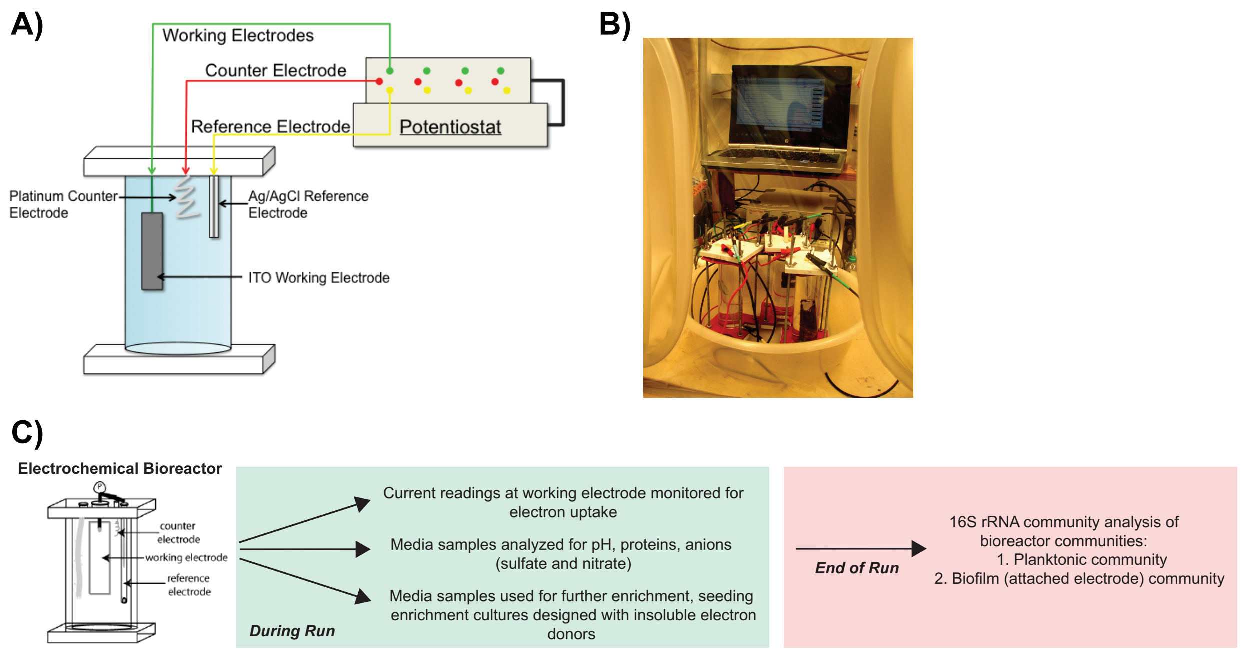

Supplementary Figure 1. Three-electrode sediment free bioreactors used for electrochemical enrichment. (A) Diagram of the three-electrode system set-up used for enrichment in the bioreactors, (B) electrochemical bioreactors incubated in the anaerobic chamber, and (C) workflow diagram of bioreactor operation.

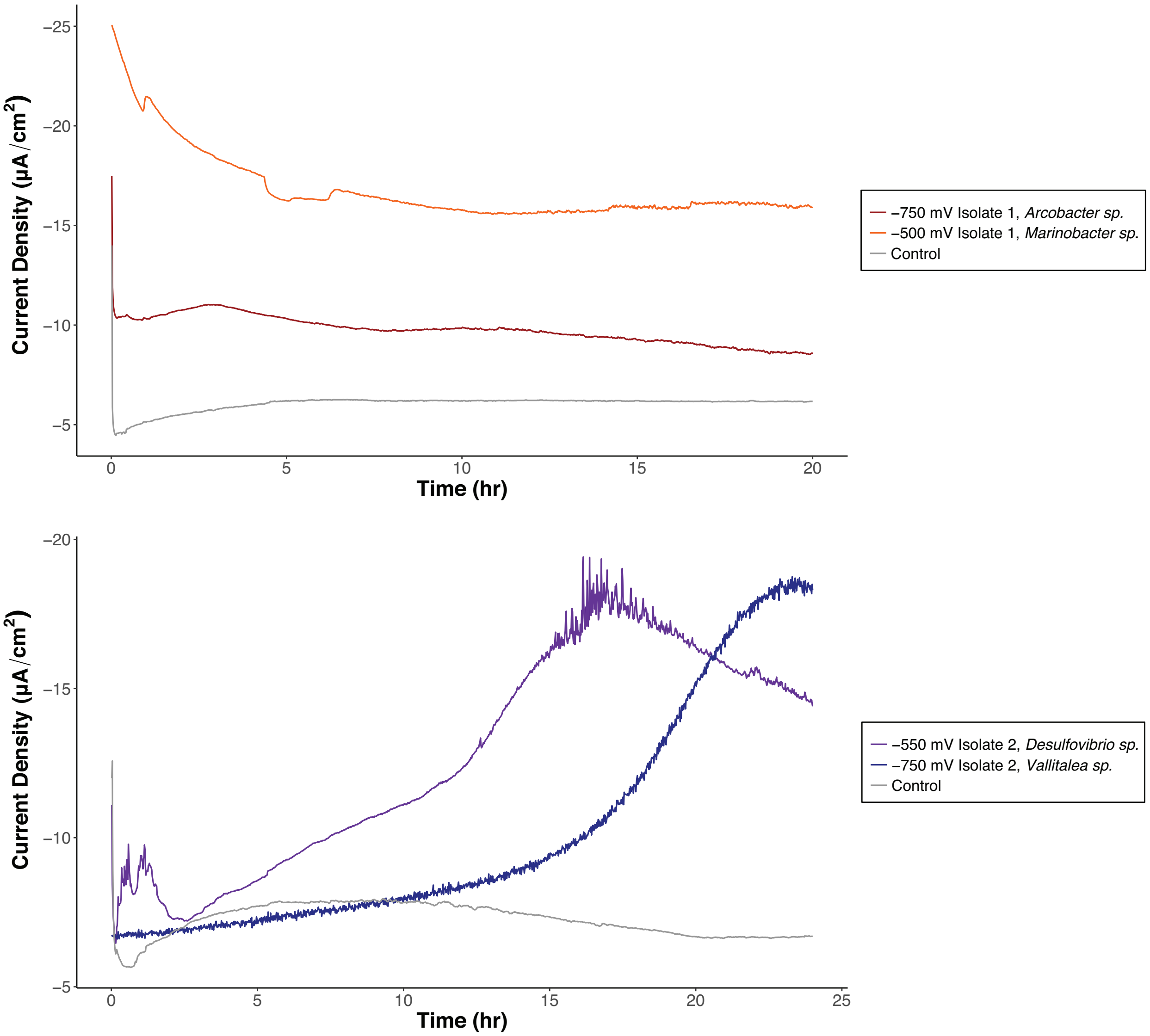

Supplementary Figure 2. Representative chronoamperometry plots for isolates over the course of 20-24 hrs compared to a control of sterile ASW. Electrodes were poised at -600 mV vs. Ag/AgCl for the -750 mV isolates and all other isolate tests were conducted at a poised potential of -400 mV vs. Ag/AgCl.

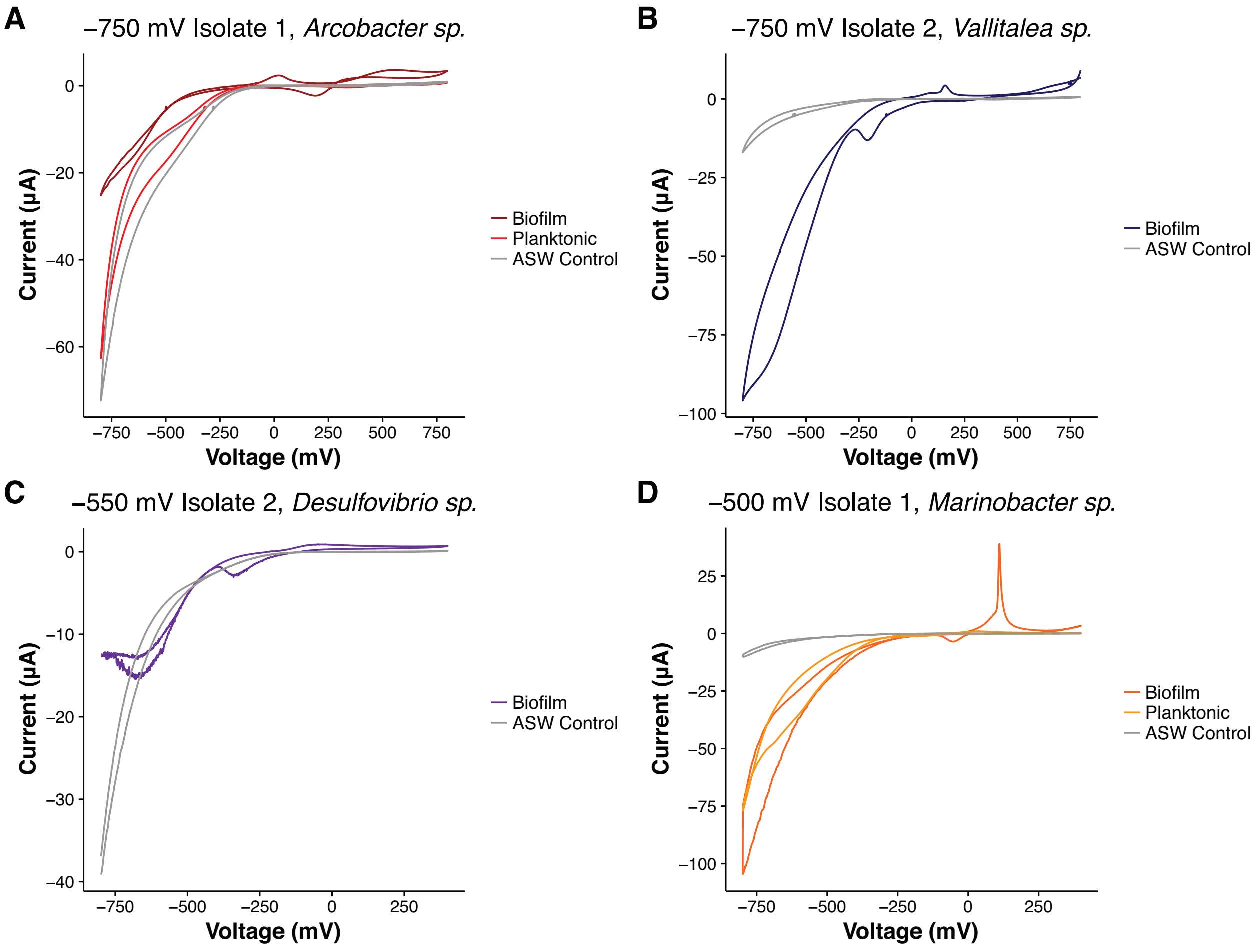

Supplementary Figure 3. Representative cyclic voltammetry plots under non-turnover conditions for isolates listed (A-D). Cyclic voltammetry was conducted on biofilm, planktonic cells, and control sterile artificial seawater (ASW control). Scans were run at 5 mV/sec.

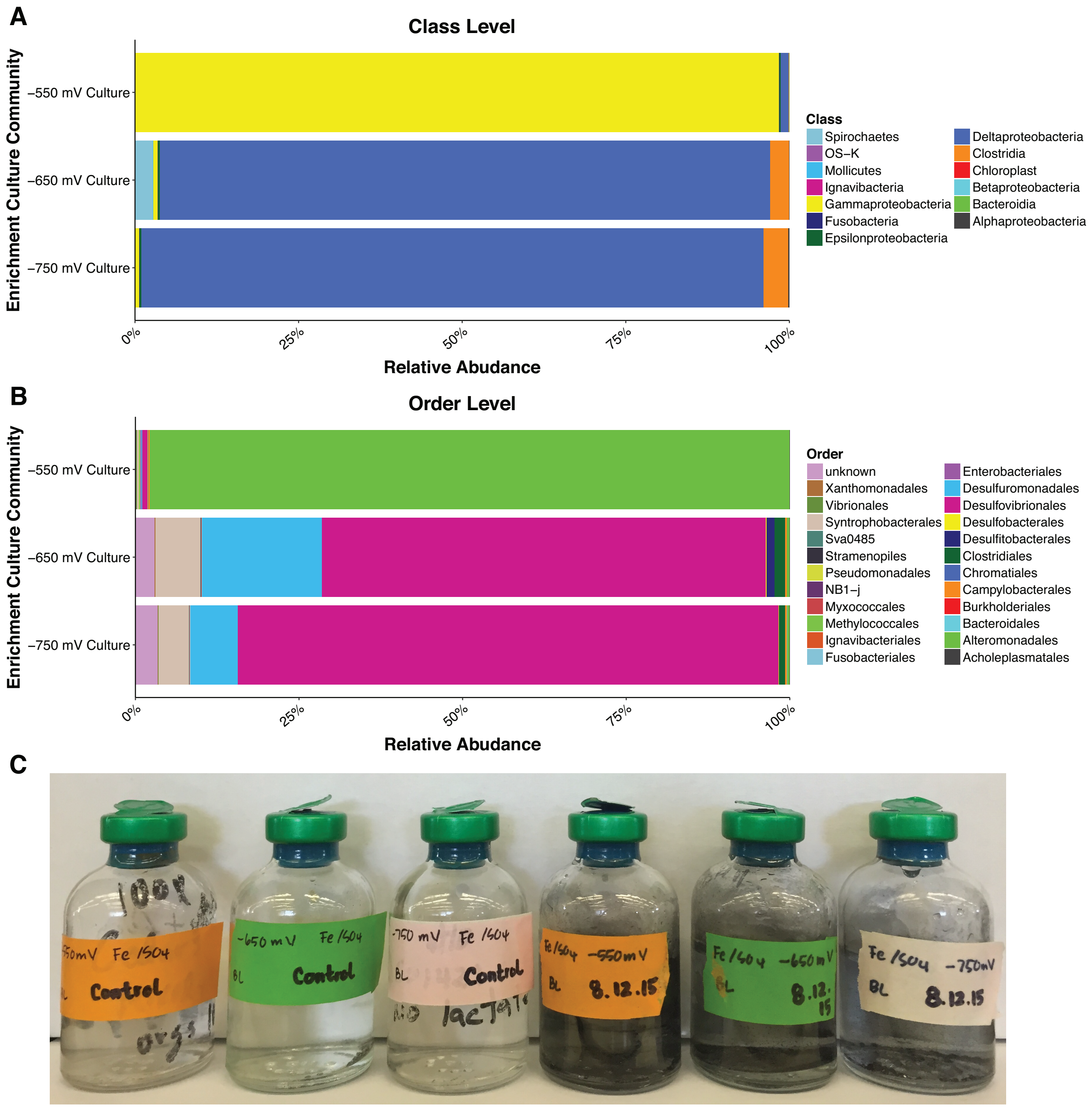

Supplementary Figure 4. Relative abundance at the (A) class and (B) order levels (top 150 OTUs) of Fe^0/SO_4 enrichment cultures, and (C) photograph of Fe^0/SO_4 enrichment culture serum vials. Enrichment cultures were initially inoculated with media from the -550, -650, and -750 mV (vs. Ag/AgCl) electrochemical bioreactors.
